# Early detection of RA‑ILD—A novel screening protocol with pulmonary function testing and lung ultrasound: A monocentric cohort study

**DOI:** 10.1007/s00393-025-01775-0

**Published:** 2026-01-23

**Authors:** Carina Fischinger, Florian Popp, Frank Reichenberger, Nikolaus Kneidinger, Robin Tiede, Werner von Wulffen, Martin Welcker

**Affiliations:** 1 Department of Pneumology, LMU University Hospital Munich, Munich, Germany; 2 Rheumacare, SYNLAB MVZ für Rheumatologie, Planegg, Germany; 3 Department of Pneumology, Augustinum Hospital Munich, Munich, Germany; 4 Clinic of Pulmonary Medicine, Seefeld-Hechendorf, Germany; 5 Department of Pneumology, Medical University Graz, Graz, Austria; 6 MVZ für Rheumatologie Dr. M. Welcker, Planegg, Germany; 7 Rheumatologie Welcker, Planegg, Germany; 8 Rheumatologie WELCKER, Bahnhofstraße 21, 82152 Planegg, Germany

**Keywords:** Seropositive rheumatoid arthritis, Interstitial lung disease, Radiation free, Pulmonary function test, Lung ultrasound, Seropositive rheumatoide Arthritis, Interstitielle Lungenerkrankung, Strahlenfrei, Lungenfunktionstest, Lungenultraschall

## Abstract

**Background:**

Seropositive rheumatoid arthritis (RA) is linked to cardiovascular and pulmonary morbidity. Nevertheless, there is currently no standardized screening method for early detection of RA-associated interstitial lung disease (ILD).

**Purpose:**

This study proposes using pulmonary function testing (PFT) combined with lung ultrasound (LUS) as additional and radiation-free method to screen for ILD inRF- and ACPA-positive RA patients. Particularly in light of the increased therapeutic options, early detection of RA-ILD is associated with better outcome.

**Methods:**

We included 214 consecutive patients with diagnosed RF- and ACPA-positive RA in our prospective study. These patients underwent PFT including spirometry, body plethysmography, and cardiopulmonary exercise testing as well as ultrasound examination of the lungs and the heart.

**Results:**

A total of 203 patients (mean age 59 ± 12 years, 24% male, 43% current or previous smokers) were examined. The overall average RA duration was 8 ± 7 years, with 32% of all patients suffering from an erosive disease course. In PFT, 60 patients (30%) showed a limitation in forced vital capacity (FVC) as well as a diffusion disorder, defined as FVC and diffusing capacity of the lung for carbon monoxide (DLCOc) ≤ 80%. Ultrasound changes were seen in 107 patients (53%), with 29% (*n* = 58) showing typical LUS patterns suspicious of ILD. In summary, ILD was suspected in almost 16% of patients (*n* = 32). With the combination of PFT and LUS, our ILD screening protocol achieves a high level of sensitivity (93%) and specificity of 72%.

**Conclusion:**

Our study contributes to the growing body of evidence supporting the use of LUS for screening for RA-associated ILD. We propose that LUS, in conjunction with PFT, serves as a suitable imaging tool for ILD screening in RA.

**Supplementary Information:**

The online version of this article (10.1007/s00393-025-01775-0) includes appendices A–E.

## Abstract (de)

**Hintergrund:**

Die seropositive rheumatoide Arthritis (RA) geht mit kardiovaskulären und pulmonalen Komorbiditäten einher. Dennoch gibt es bisher keine standardisierte Screeningmethode zur Früherkennung der RA-assoziierten interstitiellen Lungenerkrankung (RA-ILD).

**Ziel der Arbeit:**

Ziel der Studie war es, einen alternativen Ansatz vorzuschlagen, bei dem die Lungenfunktionsprüfung (PFT) kombiniert mit dem Lungenultraschall (LUS) als zusätzliche, strahlungsfreie Methode eingesetzt wird zum Screening von Rf- und ACPA-positiven RA-Patienten. Eine frühe Erkennung der RA-ILD ist mit einem deutlich besseren Outcome assoziiert.

**Methoden:**

In die prospektive Studie wurden 214 konsekutive Patienten mit diagnostizierter RF- und ACPA-positiver RA aufgenommen, die sich einer Lungenfunktionsprüfung einschließlich Bodyplethysmographie und kardiopulmonaler Belastungsprüfung sowie einer Ultraschalluntersuchung der Lunge und des Herzens unterzogen.

**Ergebnisse:**

203 Patienten (Durchschnittsalter 59 ± 12 Jahre, 24 % männlich, 43 % aktuelle oder frühere Raucher) wurden untersucht. Die gesamt-durchschnittliche Dauer der RA betrug 8 ± 7 Jahre, erosive Verläufe bei 32 % aller Patienten. Bei der PFT zeigten 60 Patienten (30 %) eine eingeschränkte FVC sowie eine Diffusionsstörung, definiert als FVC und DLCOc ≤ 80 %. Sonografische Veränderungen wurden bei 107 Patienten (53 %) festgestellt, 29 % *n* = 58) mit LUS- typischen Mustern. Bei ca. 16 % der Patienten (*n* = 32) bestand der Verdacht auf eine ILD. Die Kombination aus PFT und LUS erreichte mit 93 % eine hohe Sensitivität und eine Spezifität von 72 %.

**Schlussfolgerung:**

Diese Studie unterstützt den vielversprechenden Einsatz von LUS kombiniert mit der PFT zum Screening auf RA-ILD. Registrierung im Deutschen Register für Klinische Studien (DRKS00028871).

## Background

Rheumatoid arthritis (RA) is a chronic systemic autoimmune disease and the most common inflammatory joint disorder, with a prevalence ranging from 0.5% to 1.0% [1]. Approximately 65%–80% of RA patients are seropositive for mostly rheumatoid factor (RF; IgM) and/or ACPA antibodies. Anti-citrullinated peptide antibodies (ACPA) are highly specific for RA (sensitivity ~70%, specificity 95%) and associated with more severe disease, increased inflammatory activity, and a higher risk of extra-articular manifestations [2–4]. Pulmonary manifestations of RA include obstructive disease (1%–21%), bronchiectasis (3%–62%), pleural involvement (up to 70% postmortem), pulmonary nodules (up to 30%), and treatment-related complications [5]. Rheumatoid arthritis-associated interstitial lung disease (RA-ILD) is the most common pulmonary complication, affecting 3%–6% of RA patients, depending on the literature [6, 7]. It may present in patterns such as usual interstitial pneumonia (UIP), non-specific interstitial pneumonia (NSIP), organizing pneumonia, or diffuse alveolar damage, all of which may progress. Rheumatoid arthritis-associated ILD is associated with higher morbidity and mortality [1, 4, 8], especially with late diagnosis in advanced stages, when patients are already suffering from symptoms such as dyspnea or dry cough. Early detection is therefore crucial, particularly as new effective treatment options exist [4, 9, 10]. Disease progression is variable, and RA-ILD may even occur before joint symptoms appear.

Relevant risk factors for RA-ILD that have been mentioned in various studies [4, 5] are seropositivity, long disease duration, high disease activity, smoking, higher age, and male sex. Screening strategies for RA-ILD have been proposed, with current practice relying on clinical evaluation and pulmonary function testing (PFT), including diffusion capacity. However, PFT has only moderate diagnostic ability for detection of ILD, as shown in the study by Showalter et al. [11]. As an imaging procedure, high-resolution computed tomography (HRCT) is considered to be the gold standard for diagnosis of ILD, with detection rates between 20% and 60% in RA patients. Its use is limited by radiation exposure, cost, potential overdiagnosis, and availability in daily practice, especially in rheumatology with a large number of potentially affected patients (prevalence of RA between 0.8% and 5.5%). In this context, lung ultrasound (LUS) has emerged as a radiation-free alternative, capable of detecting subpleural interstitial changes using defined sonographic criteria [12–14]. Studies report LUS sensitivity of 73.5%–100% and specificity of 53%–97.3% compared to HRCT for diagnosing ILD, although the results may be affected by comorbidities such as infections or cardiological pulmonary congestion [15–17]. Therefore, in addition to PFT and LUS, we also performed transthoracic echocardiography (TTE) and cardiopulmonary exercise testing (CPET) in this study to rule out possible cardiac comorbidities that could have an impact on lung function.

This prospective study used LUS and PFT to screen for interstitial lung disease (ILD) in a cohort of RF- and ACPA-positive RA patients without any known pulmonary or cardiac disease.

## Methods

This monocentric, prospective, epidemiological study with a cross-sectional analysis screened asymptomatic patients with RA for the presence of ILD. In addition to PFT and LUS, the included patients underwent TTE and CPET to rule out severe cardiac comorbidities that could influence the PFT results.

A STARD-style flowchart illustrating the entire process of patient inclusion and screening procedures as well as HRCT verification and final analysis is included in the Supplementary Materials (Appendix E).

### Inclusion and exclusion criteria

We included patients with RF- and ACPA-positive rheumatoid arthritis (RA) already affiliated with a specialist outpatient rheumatology practice who met the following inclusion criteria for the purpose of screening for interstitial lung disease (ILD):

#### Inclusion criteria

- Patients diagnosed with RF- and ACPA-positive RA according to the Aletaha EULAR/ACR (European Alliance of associations for Rheumatology/American College of Rheumatology) classification criteria [18], irrespective of the time since diagnosis and the use of current or prior disease-modifying antirheumatic drugs (DMARDs).
- Absence of known or previously diagnosed cardiopulmonary diseases.
- Lack of symptoms indicative of cardiopulmonary impairment, such as cough, sputum production, breathlessness during exertion or at rest, and thoracic discomfort/chest pain.
- No lung function screening conducted within 6 months prior to study inclusion.
- Male or female participants aged ≥ 18 years at baseline.
- No evidence of active infection, specifically a negative test result for SARS-CoV‑2 (via PCR or antigen test).
- Provision of written informed consent in accordance with the International Conference on Harmonization Good Clinical Practice (ICH-GCP) guidelines and local legal requirements prior to participation in the study.

A detailed description of the exclusion criteria can be found in Supplementary Appendix A.

### Data management

Patient data were pseudonymized and securely stored in a password-protected file on a non-internet-enabled computer.

### Ethics

The study was registered in the German Register of Clinical Studies (DRKS00028871) and received ethical approval from the Ethics Committee of the University of Munich, Germany (21–1138).

### Screening protocol

After providing written informed consent, the participants underwent a structured screening process that included the following steps:

- Data collected from medical records, which documented the duration since RA diagnosis, smoking status, and current rheumatological medication as well as a comprehensive medical history and physical examination. All patients were already affiliated with the outpatient practice MVZ for Rheumatology, Dr Martin Welcker, in Planegg.
- Completion of RA-specific questionnaires assessing disease activity via scores (RAID, RADAI, CDAI, SDAI, DAS28-CRP, and DAS28-ESR) [18–21].
- Laboratory assessment of inflammatory activity, including measurements of C‑reactive protein (CRP), erythrocyte sedimentation rate (ESR), rheumatoid factor (RF), ACPA antibodies (ACPA), creatinine, and NT-pro BNP.
- Pulmonary function testing (PFT) encompassing spirometry, body plethysmography, and diffusion capacity using the methane CO method with hemoglobin correction based on the Global Lung Initiative (GLI) normal values.
- Cardiopulmonary exercise testing (CPET) conducted on a treadmill in an upright body position, incorporating 12-lead ECG monitoring, analysis of expiratory gas samples using a CPET mask, blood gas analysis at rest and during exercise from arterialized earlobe samples, oxygen saturation, and non-invasive blood pressure measurements. The exercise protocol included a 1-min preload phase followed by a ramp protocol designed to achieve maximum workload within approximately 10 min, adjusted to the participant’s physical condition. The anaerobic threshold (AT) was determined using the V‑slope method.
- Transthoracic pleuropulmonary ultrasound (LUS) using a convex 3.5 MHz probe (Hitachi Aloka ProSound Alpha 6 or Hitachi Arietta V70) in an upright position following a predefined protocol examining dorsal, lateral, and ventral thoracic views at 14 specified scanning points; LUS was conducted by two of three investigators (F.R., M.H., W.v.W.) in consensus.
- Transthoracic echocardiography (TTE) performed according to routine clinical protocols to exclude significant cardiac comorbidities.
- Resting and exercise 12-lead electrocardiograms (ECG).

### Definition criteria for suspected ILD

An ILD suspicion was defined as impaired PFT results, specifically DLCOc-SB ≤ 80% predicted or FVC ≤ 80% predicted, in combination with sonographic evidence of ILD-like interstitial changes on LUS in the absence of other abnormal cardiopulmonary findings during screening.

As sonographic signs of ILD, we applied pleural line fragmentation and pleural thickening as well as the presence of > 5 hyperechoic vertical artifacts (B-lines) per field of view [17, 22, 23]. For a detailed screening protocol and sample images, please refer to the publication by Reichenberger et al. [24].

Patients with notable LUS findings, such as indications of interstitial lung disease (ILD) or consolidations, were recommended to undergo further diagnostic tests outside of the study as part of routine follow-up, including high-resolution computed tomography (HRCT) as gold standard.

### Statistics

The statistical analysis was performed by using SPSS version 30.0 (IBM Corp., Armonk, NY, USA) and Microsoft Excel version 16.96.1 (Microsoft Corporation, Redmond, WA, USA) for Mac OS.

The results are presented as mean ± standard deviation (SD). Subgroup comparisons were conducted using 95%CI to evaluate the relevance and significance of the differences.

We used receiver operating characteristic (ROC) analysis and the area under the curve (AUC) to estimate the value of the different combinations of tests regarding screening with LUS and PFT—alone or in combination. The optimal cut-off value for the individual tests was determined using the Youden index. Indications of sensitivity and specificity are provided by the four-field tables.

## Results

### Cohort description

Between April 2022 and January 2025, a total of 214 consecutive patients with rheumatoid arthritis (RA) who met the inclusion criteria and provided written informed consent were enrolled in the study. Of these, 11 patients withdrew their consent for personal or health reasons or due to repeated scheduling conflicts. As a result, 203 patients underwent examinations as part of our study.

At the time of inclusion in the study, none of the patients reported respiratory symptoms such as dyspnea at rest or on exertion, cough, or chest pain in their medical history.

Patients in the overall cohort were characterized by a mean age of 59.2 ± 12.2 years, a mean RA duration of 8.3 ± 7.2 years, a male proportion of 24.1%, and 43.3% smokers, as shown in Table 1. Of all patients, 31.5% had an erosive RA disease course, while the mean values of disease activity scores and inflammatory parameters (CRP and ESR) were in the low range. The overall cohort showed a mean rheumatoid factor score of 128.9 ± 190.9 and a mean ACPA antibody score of 208.0 ± 108.3. We divided the total cohort into two groups: patients with suspected ILD (susILD, *n* = 32) and patients without ILD suspicion (nonILD, *n* = 171).

**Table 1:** Descriptive representation of data

	Total cohort	susILD group	nonILD group	Subcohort HRCT
	*N* = 203 (100.0%)	*n* = 32 (15.8%)	*n* = 171 (84.2%)	*n* = 43 (21.2%)
*Anamnestic data*				
Age, years	59.2 ± 12.2	64.3 ± 12.9	58.3 ± 11.9	62.7 ± 10.9
Male,* n* (%)	49 (24.1%)	9 (28.1%)	40 (23.4%)	11 (25.6%)
BMI, kg/m^2^	25.7 ± 4.6	26.1 ± 4.5	25.6 ± 4.7	25.4 ± 4.8
Previous or current smoking,* n* (%)	88 (43.3%)	20 (62.6%)	68 (39.7%)	28 (65.1%)
Packyears	10.7 ± 16.3	20.2 ± 20.6	8.9 ± 14.8	19.9 ± 20.4
Disease duration (RA), years	8.3 ± 7.2	8.8 ± 6.7	8.2 ± 7.3	8.5 ± 7.1
Erosive,* n* (%)	64 (31.5%)	18 (56.3%)	46 (26.9%)	23 (53.5%)
csDMARDs,* n* (%)	122 (60.1%)	18 (56.3%)	104 (60.8%)	29 (67.4%)
tsDMARDs,* n* (%)	36 (17.7%)	3 (9.4%)	33 (19.3%)	6 (14.0%)
bDMARDs,* n* (%)	101 (49.8%)	18 (56.3%)	83 (48.5%)	23 (53.5%)
Steroids,* n* (%)	21 (10.3%)	3 (9.4%)	18 (10.5%)	6 (14.0%)
*Scores testing the activity of the RA*				
DAS28 CRP	2.3 ± 0.9	2.4 ± 0.9	2.2 ± 0.9	2.5 ± 0.9
DAS28 ERS	2.4 ± 1.1	2.6 ± 1.1	2.3 ± 1.1	2.6 ± 1.1
RADAI	1.9 ± 1.5	2 ± 1.3	1.9 ± 1.5	2.4 ± 1.6
RAID	2.4 ± 2.0	2.5 ± 1.7	2.3 ± 2	2.8 ± 1.8
CDAI	5.7 ± 6.2	6.8 ± 6.1	5.5 ± 6.2	7.7 ± 7.4
SDAI	6.2 ± 6.3	7.3 ± 6.2	6 ± 6.3	8.2 ± 7.5
*Laboratory parameter*				
ESR, mm/h	16.4 ± 13.6	16.7 ± 12	16.3 ± 13.9	16.8 ± 14.3
CRP, mg/l	3.9 ± 4.4	4.8 ± 3.5	4.4 ± 4.6	4.4 ± 3.2
Rheumatoid factor, IU/ml	128.9 ± 190.9	108.3 ± 101.6	132.8 ± 203.3	141.8 ± 213.1
CCP antibodies, IU/ml	208.0 ± 108.3	211.5 ± 113.2	207.3 ± 107.7	215.8 ± 113.0
Creatinine, mg/dl	0.8 ± 0.2	0.8 ± 0.2	0.8 ± 0.1	0.8 ± 0.2
Hemoglobin, g/dl	13.9 ± 1.1	13.9 ± 1.2	13.9 ± 1.1	13.8 ± 1.0
BNP, pg/ml	129.0 ± 142.4	193.0 ± 171.2	131.0 ± 133.6	142.1 ± 140.3

Data are presented as mean with standard deviation or as number (*n*)

*susILD* suspected ILD, *nonILD* no ILD-suspicion, *ILD* interstitial lung disease, *BMI* body-mass-index, *csDMARDs* conventional synthetic disease-modifying antirheumatic drugs, *tsDMARDs* targeted synthetic disease-modifying antirheumatic drugs, *bDMARDs* bological disease-modifying antirheumatic drugs, *DAS* disease activity score, *CRP* c-reactive protein, *ERS* erythrocyte sedimentation rate, *RADAI* rheumatoid arthritis disease activity index, *RAID* rheumatoid arthritis impact of disease, *CDAI* clinical disease activity index, *SDAI* simple disease activity index, *CCP* cyclic-citrullinated peptide, *BNP* brain natriuretic peptide

Table 1 shows the basic parameters of the two subcohorts, susILD and nonILD, as mean values with standard deviations or as number (*n*) with percentage share of the respective subcohort.

The susILD cohort had a slightly higher age than the nonILD cohort (64 ± 12 years [95%CI 60; 69] vs. 58 ± 12 years [95%CI 57; 60]) as well as a higher relative proportion of men (28% vs. 23%). In the susILD cohort, higher packyear values were noticeable (20 ± 21 packyears [95%CI 13; 28] in the susILD group vs. 9 ± 15 packyears [95%CI 7; 11] in the nonILD group) as well as considerably more patients with a positive smoking history as current or former smokers (63% susILD vs. 40% nonILD). A detailed table with confidence intervals can be found in Appendix B.

While both subcohorts had comparable mean values for BMI and duration of RA since diagnosis, there were more patients in the susILD group who had an erosive course of RA (56.3% vs. 26.9%).

Regarding RA disease activity, the mean DAS28-CRP, DAS28-ERS, RADAI, and RAID values were comparable between the groups, while the mean CDAI (6.8 ± 6.1 vs. 5.5 ± 6.2) and SDAI (7.3 ± 6.2 vs. 6.0 ± 6.3) were slightly higher in the susILD group (Table 1).

No significant increases in inflammatory activity in the laboratory inflammatory parameters were described. As only patients with RF and ACPA antibodies were included in this study, both antibodies were in the detectable positive range. The mean value for rheumatoid factor was slightly higher in the nonILD group, while the mean value for the ACPA antibody hardly differed between the two groups.

The mean BNP value was slightly higher in the subcohort with suspected ILD, at 193 ± 171 pg/ml compared to 117 ± 134 pg/ml in the nonILD group.

### Lung function testing

#### Pulmonary function testing and cardiopulmonary exercise testing

All participants underwent pulmonary function testing (PFT). Across the cohort, the mean FEV1 was 95.3 ± 18.1% [92.8; 97.8], mean FVC was 97.3 ± 16.9% [95.0; 99.7], and mean TLC was 105.7 ± 14.8%. Diffusion capacity measurements showed a mean DLCOCc-SB of 80.2 ± 15.6%. The susILD group showed worse results than the nonILD group, especially in terms of FEV1 90.8% (± 19%), FVC 94.6% (± 16.2%) and DLCOc 68.2% (± 13.5%) (Table 2).

**Table 2:** Results of the various studies

	Total cohort *N* = 203 (100.0%)	susILD group *n* = 32 (15.8%)	nonILD group *n* = 171 (84.2%)	Subcohort HRCT *n* = 43 (21.2%)
*Lung function testing*				
FEV1, %	95.3 ± 18.1	90.8 ± 19	96.2 ± 17.9	87.4 ± 20.9
FVC, %	97.3 ± 16.9	94.6 ± 16.2	97.9 ± 17.0	94.7 ± 19.1
DLCOc, %	80.2 ± 15.6	68.2 ± 13.5	82.4 ± 14.9	70.5 ± 15.8
Tiffeneau	0.8 ± 0.1	0.8 ± 0.1	0.8 ± 0.1	0.7 ± 0.1
TLC, %	105.7 ± 14.8	105.1 ± 13.8	105.8 ± 15.0	104.2 ± 15.1
RV, %	117.5 ± 32.4	121.6 ± 27.8	116.7 ± 33.2	126.1 ± 37.5
*Cardiopulmonary exercise testing*				
Peak work rate, watts	127.8 ± 44.5	109.6 ± 36.4	131.3 ± 45.2	110.8 ± 37.9
Predicted work rate, watts	120.3 ± 37.1	105.1 ± 41.1	123.2 ± 35.6	109.3 ± 38.0
VO_2_ peak, %	97.3 ± 23.7	86.9 ± 23.0	99.3 ± 23.4	91.6 ± 23.0
VO_2_ AT, %	79.5 ± 23.0	71.8 ± 22.5	81.0 ± 22.9	76.9 ± 22.7
EQCO_2_ slope	29.6 ± 6.1	32.3 ± 5.5	29.1 ± 6.1	31.6 ± 5.9
pO_2_ peak, mm Hg	84.7 ± 11.8	82.7 ± 13.1	85.1 ± 11.6	83.1 ± 13.5
pCO_2_ peak, mm Hg	35.5 ± 4.6	34.1 ± 5.6	35.8 ± 4.4	35.1 ± 5.3
*Lung ultrasound*				
LUS sPAP, mm Hg	24.1 ± 6.9	26.5 ± 7.5	23.8 ± 6.7	25.3 ± 7.4
LUS TAPSE, mm	24.0 ± 3.8	24.4 ± 3.9	24.0 ± 3.8	24.2 ± 3.6
LUS LV_EF, %	65.8 ± 6.1	67.6 ± 3.9	65.5 ± 6.4	65.7 ± 5.5
*Evaluation lung function testing*				
Abnormalities in total,* n* (%)	82 (40.4%)	30 (93.8%)	52 (31.1%)	33 (78.6%)
Diffusion disorder,* n* (%)	58 (28.6%)	27 (84.4%)	31 (18.6%)	27 (64.3%)
Limitation in FVC,* n* (%)	2 (1.0%)	2 (6.3%)	0 (0.0%)	1 (2.4%)
Restriction,* n* (%)	13 (6.4%)	2 (6.3%)	11 (6.6%)	4 (9.5%)
Obstruction,* n* (%)	26 (12.8%)	9 (28.1%)	17 (10.2%)	14 (33.3%)
Hyperinflation,* n* (%)	8 (3.9%)	2 (6.3%)	6 (3.6%)	4 (9.5%)
PostOP gentle respiration,* n* (%)	3 (1.5%)	1 (3.1%)	2 (1.2%)	1 (2.4%)
*Evaluation lung ultrasound*				
Abnormalities in total,* n* (%)	107 (52.7%)	32 (100.0%)	75 (44.1%)	36 (83.7%)
ILD,* n* (%)	58 (28.6%)	31 (96.9%)	27 (15.9%)	26 (60.5%)
Pleural irregularities,* n* (%)	26 (12.8%)	0 (0.0%)	26 (15.3%)	4 (9.3%)
Comet tail artefacts/B-lines,* n* (%)	63 (31.0%)	25 (78.1%)	38 (22.2%)	21 (48.8%)
Pleural effusion,* n* (%)	14 (6.9%)	4 (12.5%)	10 (5.9%)	5 (11.6%)
Pulmonary congestions,* n* (%)	2 (1.0%)	0 (0.0%)	2 (1.2%)	0 (0.0%)
Focal changes/tumor,* n* (%)	16 (7.9%)	3 (9.4%)	13 (7.6%)	7 (16.3%)
Others,* n* (%)	5 (2.5%)	1 (3.1%)	4 (2.4%)	1 (2.3%)
*Evaluation ILD suspicion*				
Based on LUS,* n* (%)	65 (32.0%)	31 (96.9%)	34 (20.0%)	26 (60.5%)
Based on *lung function testing* and LUS,* n* (%)	32 (15.8%)	32 (100.0%)	0 (0.0%)	21 (48.8%)

Data are presented as mean with standard deviation or as number (*n*)

Among the patients, 26 exhibited an obstructive airway pattern (FEV1/FVC < 70%) and 13 demonstrated a restrictive pattern (TLC < 80%), affecting more patients of the nonILD group (obstruction 17, 10.2%; restriction 11, 6.6%). Moreover, 27 of 32 susILD patients (84.4%) and 31 of 171 nonILD patients (18.6%) reached a DLCOCc-SB < 80%, consistent with a diffusion disorder (Table 2).

#### Cardiopulmonary exercise testing

In addition to PFT, CPET was performed to detect possible, particularly cardiac, comorbidities that may have an impact on lung function and thus the study results. Regardless of the subcohort, every subcohort achieved a higher peak workload than the predicted work rate in CPET. The mean peak VO_2_ in the nonILD group was 99.3 ± 23.4%, while the susILD cohort showed considerably lower values (86.9 ± 23.0%). Also, the anaerobic threshold (AT) was lower in the susILD group than in the nonILD group (71.8 ± 22.5% vs. 81.0 ± 22.9%). The mean slope of the ventilatory equivalent for CO_2_ (EQCO_2_) was 29.6 ± 6.1. None of the patients developed hypoxemia or exhibited signs of cardiac ischemia during or after exercise (Table 2).

### Ultrasound testing

#### Lung ultrasound

Lung ultrasound was conducted on each patient according to the study protocol. Of all participants, 52.7% (*n* = 107) showed abnormalities in lung ultrasound (LUS), 32 (100%) patients of the susILD group and 75 (44.1%) of the nonILD-group (Table 2).

Signs suggestive of potential ILD, like bilateral pleural fragmentation (*n* = 26), pleural thickening (*n* = 19), and comet tail artefacts as well as > 5 B‑lines (*n* = 63), could be identified. Additionally, small pleural effusions not suitable for pleurocentesis were observed in 16 cases (7.9%).

One patient exhibited bilateral fragmentation, pleural thickening, and B‑lines, along with pleural effusion, renal dysfunction, and cardiac abnormalities on transthoracic echocardiography (TTE). These findings were attributed to fluid overload in the context of cardiorenal compromise and were not related to ILD.

#### Transthoracic echocardiography

To exclude significant cardiac comorbidities, we performed TTE. The mean left ventricular systolic function was physiological at 65.8 ± 6.1%, independent of the subcohort, while the mean systolic pulmonary artery pressure (sPAP) corrected for central venous pressure (CVP) was 24.1 ± 6.9 mm Hg, and the mean right ventricular tricuspid annular plane systolic excursion (RV-TAPSE) was 24.0 ± 3.8 mm. There were no significant differences between the susILD group and the nonILD group.

Severe aortic stenosis requiring intervention was identified in one patient with a bicuspid aortic valve. Other findings included mild diastolic compliance disorder (*n* = 33), left ventricular hypertrophy (*n* = 9), aortic stenosis (*n* = 5), minimal mitral valve prolapse (*n* = 1), and signs of coronary heart disease (*n* = 2).

No evidence of pulmonary vascular involvement was observed in any patient.

#### Suspicion of ILD in the context of the test results

Impaired pulmonary function testing (PFT) results were exhibited by 60 patients (29.6%), characterized by DLCOc-SB/KCOc-SB ≤ 80% predicted and FVC < 80%. Among these, in 32 patients (15.8%), a pattern suspicious of interstitial lung disease (ILD) was identified via lung ultrasound (LUS).

This subset of patients was classified as having suspected ILD (susILD), representing almost 16% of the total cohort.

#### Comparison between the suspected ILD and non-ILD groups

The previously described risk factors [4, 5] are also evident in the results of our study when comparing the two subcohorts: the susILD group shows higher age, relatively more male patients, more previous and current smokers as well as a significantly greater cumulative smoking exposure (packyears), and relatively more patients with an erosive course. Comparison of lung function revealed that the susILD group had a considerably reduced diffusion capacity and diminished exercise capacity, as measured by the VO_2_ peak at maximum exertion and VO_2_ at the anaerobic threshold (AT) during cardiopulmonary exercise testing (CPET). The groups exhibited comparable distributions of lung volume and indices of rheumatic disease activity. Patients in the susILD group were predominantly treated with monotherapy (71.9%) and bDMARDs (56.3%), whereas the nonILD group received monotherapy (60.8%) and bDMARDs (48.5%) less frequently. Our results suggest that tsDMARDs have a protective effect on the development of RA-ILD (tsDMARDs 9.4% susILD vs. 19.3% nonILD), which is consistent with the trend reported in a 2023 study [25]. However, further data are needed to support this hypothesis.

Tables 1 and 2 show the detailed data.

### High-resolution computed tomography

#### Follow-up as per gold standard

Patients with suspicious findings on LUS were recommended to undergo further diagnostic procedures outside of the study. The same was true for patients with a significant smoking history of up to 15 packyears who qualified for lung tumor screening according to according to the guideline [26]. Therefore, 30 patients were offered low-dose thoracic computed tomography (TCT) outside of the study.

**Fig. 1:**
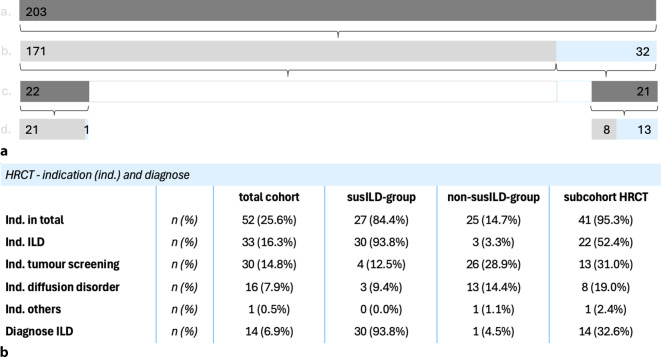
**a** *a.* Total cohort; *b.* classification into non-ILD group (group with our ILD suspicion; light gray) and suspected ILD group (group with suspected ILD; light blue) based on the screening results of pulmonary function testing and lung sonography; *c.* patients who underwent HRCT examination during follow-up; *d.* HRCT findings non-ILD (light gray) and ILD (light blue). True-to-scale representation, number *n* in figures. **b** HRCT indications (ind.) and HRCT-confirmed ILD diagnosis

In total, HRCT was performed in 43 patients during follow-up (*n* = 21 susILD and *n* = 22 nonILD) (Fig. 1).

All HRCT examinations were requested without contrast medium as standard and were based on current radiological standards for the detection of interstitial lung diseases. A total of 3 patients underwent HRCT with contrast medium. In addition, low-dose HRCT with contrast medium was used in 7 patients, in each case at the discretion of the treating radiologist in private practice.

A STARD-style flowchart illustrating the entire process of patient inclusion and screening procedures as well as HRCT verification and final analysis is included in Supplementary Appendix E.

#### Comparison between the total cohort and the subcohorts receiving HRCT and TCT

Compared to the overall cohort, the cohort that received HRCT was primarily characterized by significantly higher nicotine consumption (10.7 ± 16.3 vs. 19.9 ± 20.4 packyears). The group also contains relatively more patients with erosive disease progression, increased rheumatoid factor and CCP antibodies, and discretely increased values in the scores for measuring disease activity compared to the total cohort and the susILD group (Table 1). To identify independent predictors of ILD on HRCT and to assess whether LUS remains significant after adjustment, we performed multivariable logistic regression including age, sex, packyears, erosive disease, RA duration, and LUS findings. The overall model was highly significant (χ^2^ (6) = 27.83, *p* < 0.001), with good explanatory power (Nagelkerke R^2^ = 0.67) and classification accuracy (sensitivity 85.7%, specificity 93.1%). Among the covariates, only packyears showed an independent association with ILD (adjusted OR 0.93 per packyear, *p* = 0.032), whereas age, sex, erosive disease, and RA duration were not significant.

Similar to the susILD group, the lung function tests showed a reduced FVC and DLCOc as well as a significantly increased residual volume. In summary, 64.3% of patients (*n* = 27) were found to have a diffusion and 33.3% (*n* = 14) an obstruction disorder. The indication for HRCT was primarily due to suspected ILD (*n* = 22) and a diffusion disorder in 19% (*n* = 8), as well as for TCT routine tumor screening in patients with high nicotine consumption (*n* = 13).

#### Comparison of test accuracy: novel PFT/LUS screening method versus the gold standard (HRCT)

The screening performance of LUS and PFT criteria for detecting ILD was evaluated against ILD findings in HRCT in the subcohort with HRCT. The combined use of lung ultrasound (LUS) and pulmonary function testing (PFT) criteria demonstrated a diagnostic sensitivity of 93%, specificity of 72%, PPV of 62%, and NPV of 95%.

**Fig. 2:**
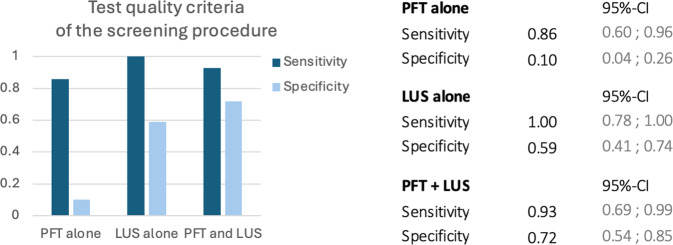
Test quality criteria of the screening procedure based on pulmonary function testing *(PFT*) alone, lung ultrasound (*LUS*) alone, and a combination of PFT and LUS compared to ILD diagnosis by HRCT as the gold standard (*n* = 43). The 95% confidence intervals (*95%CI*) are included. For more details, please refer to Appendix C

Applying LUS positivity alone, the diagnostic performance yielded with 100% a higher sensitivity and NPV, while the specificity and PPV decreased to 59% and 54%, respectively. Using lung function alone with the criteria FVC or DLCOc ≤ 80%, the diagnostic performance of the tests is remarkably poorer, with a sensitivity of 86%, specificity of 10%, PPV of 32%, and NPV of 60% (Fig. 2 and Appendix C).

**Fig. 3:**
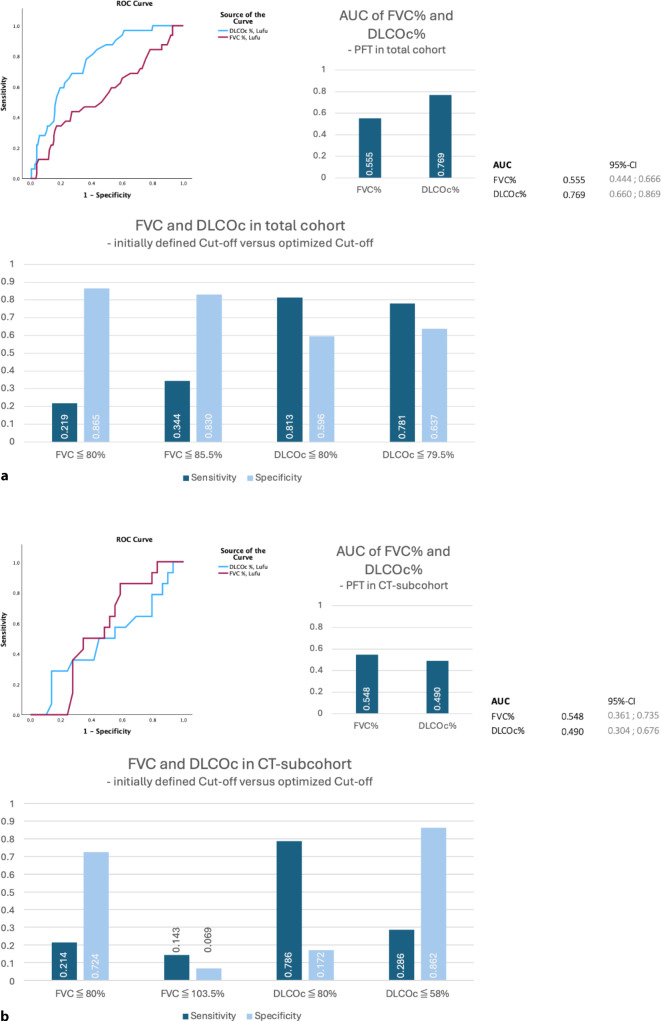
ROC analysis showing the AUC and Youden index for FVC and DLCOc based on the screening method used for the entire cohort (*n* = 203; **a**) compared with the HRCT subcohort (*n* = 43; **b**). The individual four-field tables and the 95% confidence intervals can be found in Appendix D

The ROC analysis of FVC % and DLCOc % in relation to suspicion of ILD revealed an area under the curve (AUC) of 0.555 (FVC %) and 0.769 (DLCOc %), similar to the AUC in relation to HRCT-diagnosed ILD (0.548 and 0.490; Fig. 3; for more details refer to Appendix D).

## Discussion

The development of ILD in rheumatoid arthritis (RA) represents a clinically significant manifestation with therapeutic implications. In order to achieve early detection of RA-ILD in affected patients, the relatively low prevalence requires widespread routine screening. Abnormal parameters such as higher age, male gender, smoking history, erosive course, and RF and ACPA positivity have been suggested as risk factors and as an indication of patients who are more likely to develop RA-ILD. Therefore, screening patients with positive risk factors routinely in the first instance and early course of the disease is to be considered [4, 5]. However, since HRCT—the current gold standard for diagnosing ILD—is associated with a non-negligible radiation exposure and high costs in annual screenings, additional screening procedures are needed.

In this study, as a radiation-free screening strategy, we used a combination of functional assessments and imaging, as has been increasingly recommended for ILD screening in rheumatic diseases [12–14, 27].

We performed screening of 203 consecutive RF- and ACPA-positive RA patients with the combined approach of PFT and LUS, of whom 43 participants underwent HRCT for further evaluation (21 belonged to the susILD group and 22 to the nonILD-group). We identified 32 patients with an ILD-compatible pattern (16% susILD). Figure 2 shows the differences in the test performance of the combined screening (PFT and LUS), LUS alone, and PFT alone. It should be emphasized that screening with PFT alone shows a significantly poorer test quality, which is related to the fact that PFT alone is too unspecific for RA-ILD. This is supported by a 2018 study in which ILD was detected in SSc patients using HRCT despite normal lung function tests focusing on FVC and DLCOc limitations [11].

Lung ultrasound alone, on the other hand, impresses with a higher sensitivity, but detects significantly more patients not affected by ILD as ILD patients, who subsequently receive an HRCT recommendation (specificity 59%). It is therefore associated with higher financial and time expenditure as well as with increased emotional stress for patients. Given the broad differential diagnosis, LUS findings should always be interpreted in the clinical context. In this regard, in addition to PFT and LUS, TTE and CPET analyses were also performed to rule out severe cardiac comorbidities that could influence the lung function results and to facilitate the interpretation of the LUS findings.

With the combination of both methods, our screening protocol achieves a high level of sensitivity (93%), which is associated with only few false-negative patients and a lower level of specificity (72%).

The consequence is that the combination of PFT and LUS offers the possibility of excluding patients without ILD with a high probability while at the same time detecting affected patients who subsequently require HRCT to confirm the diagnosis.

Using this method, we were able to detect more affected patients (16%) than assumed based on the current literature in a clinical setting in which attention is already focused on the possible presence of RA-ILD [6, 7].

In a few patients, the results of screening did not match those of HRCT.

One patient showed ILD-typical patterns in LUS but had no changes in the lung function tests, which is why she was added to the nonILD group in the summary of the results. This patient had no smoking history (0 packyears), had non-erosive RA, only 3 years of RA disease duration, and no symptoms. She showed discretely elevated signs of active RA in the scores (DAS28-CRP 5.2, DAS28-ERS 5.1, CDAI 35, SDAI 35.4, RADAI 6.3, RAID 5.5), but neither inflammatory parameters nor particularly high positive values for RF and ACPA in the laboratory (ERS 8 mm/h, CRP 4 mg/l, RF 10 IU/ml, ACPA 107 IU/ml). This may be a sign that even small changes in LUS may precede limitations in PFT.

In 8 patients of the susILD group, ILD was excluded by follow-up HRCT. Those patients all showed high nicotine consumption (packyears) and changes in PFT (mainly signs of diffusion disturbance, *n* = 7, and obstruction, *n* = 5) as well as comet tail artefacts and an ILD pattern in LUS. The remaining parameters recorded did not reveal any clustering.

Furthermore, 4 of the 8 patients were found to have pulmonary emphysema on HRCT. Rheumatoid arthritis-associated emphysema has already been described in earlier studies [28]. Interstitial lung disease was suspected on the basis of changes in the PFT, in particular a reduction in the FVC % and DLCOc-SB %, in addition to typical ILD patterns in the LUS. Our initially defined cut-off value was ≤ 80%.

Although diffusion capacity (DLCOc-SB) is a conventional marker for early lung involvement in rheumatic diseases, its low AUC (0.490) compared to HRCT-confirmed ILD highlights its limitations in this context. This could be related to the small CT subcohort (*n* = 43), considering that AUC gives significantly better results (0.769) in relation to suspicion of ILD.

The FVC appears to be less suitable, as both AUC in relation to the results of the screening and the HRCT are 0.555 and 0.548. Even analyzing the data using the Youden index with the aim of finding an optimum cut-off value did not produce any better values (Fig. 3; Appendix D). Accordingly, FVC is not appropriate as the sole parameter for confirming the diagnosis with this screening protocol, but it is nevertheless useful as a progression parameter and for making a prognosis.

The results of this study show a remarkable correlation between higher age, erosivity, and smoking history and susILD, supporting the established role of these factors as risk factors for ILD in RA (Table 1). There was also a discretely higher relative proportion of male patients in the susILD group (male sex: 28.1% susILD vs. 23.4% nonILD), consistent with the risk factors already mentioned. There were no significant differences between RF and ACPA levels in serum and the two subcohorts (susILD and nonILD), with only positive patients included in the study.

No major differences were found between the two groups in terms of disease activity indices (Table 1) or laboratory inflammation markers, which is related to the fact that these parameters were collected at the time of inclusion in the study, and no data are available on the time of peak RA activity during the course of the each individuals’ respective disease progression.

Regarding RA disease activity, the mean DAS28-CRP, DAS28-ERS, RADAI, and RAID indicate remission, while the mean CDAI (6.8 ± 6.1 susILD vs. 5.5 ± 6.2 nonILD) and the mean SDAI (7.3 ± 6.2 susILD vs. 6.0 ± 6.3 nonILD) both reflect low disease activity [19, 20]. This deviation could be explained by the fact that the CDAI and SDAI scores also include the physician’s assessment, which increases the sensitivity for low residual activity.

As mentioned above, the mean BNP value was slightly higher in the subcohort with suspected ILD (193 ± 171 pg/ml) compared to the nonILD group (117 ± 134 pg/ml).

This notable difference may indicate an increased burden on the heart, for example due to incipient changes in the lung parenchyma in the context of RA-ILD, but may also be related to the higher nicotine consumption and slightly higher age [29, 30].

To identify independent predictors of ILD on HRCT and to assess whether LUS remains significant after adjustment, we performed multivariable logistic regression including age, sex, packyears, erosive disease, RA duration, and LUS findings. In the multivariable logistic regression analysis, only the number of packyears remained independently associated with ILD on HRCT. However, it should be noted that the HRCT subcohort consists of patients with suspected ILD as well as patients with a high number of packyears, which distorts the results.

For the binary LUS variable, the model did not converge due to quasi-complete separation (very large coefficient and standard error), indicating that LUS almost perfectly discriminated ILD-positive from ILD-negative cases in our dataset. As a result, confidence intervals are unreliable. This suggests a strong unadjusted effect of LUS, but a valid estimation of its adjusted odds ratio requires penalized or exact logistic regression, which is not natively available in SPSS.

Secondary findings included pleural consolidation (12.8%), suspected malignancy, and pleural effusion (6.9%) on LUS. In PFT, they included impaired diffusion capacity (28.6%), partly due to pulmonary emphysema (9 patients among the 43 who underwent HRCT), a well-known comorbidity in RA patients [28], and obstructive lung disease (12.8%), which were likely influenced by the high prevalence of former and active smokers. One non-smoking patient was diagnosed with non-small cell lung cancer (NSCLC), which was amenable to curative surgical intervention. Lung cancer screening is based on HRCT in accordance with current guidelines, extending beyond the scope of LUS.

Several cardiac comorbidities were also detected, including diastolic compliance disorder (*n* = 33), left ventricular hypertrophy (*n* = 33), severe aortic stenosis (*n* = 5), pericardial effusion (*n* = 1), and signs of coronary heart disease (*n* = 2). None of the patients demonstrated evidence of pulmonary hypertension (PH), aligning with the reported low risk of PH in RA.

This study has several limitations. We only included seropositive patients with rheumatoid arthritis (RA). The focus on this cohort is justified by the fact that no studies to date have specifically investigated the prevalence of ILD in RF- and ACPA-positive RA. However, seropositive forms of the disease are associated with a higher risk of extra-articular manifestations, particularly ILD; RF and ACPA positivity are considered established risk factors in this context.

In addition, current literature indicates that RF- and ACPA-positive forms of RA are more frequently associated with a more aggressive disease course and, thus, carry an increased risk of systemic organ manifestations, including ILD. This patient group is therefore of particular clinical importance with regard to early diagnosis and targeted therapeutic interventions to improve long-term outcomes.

The aim of the study was to demonstrate the potential of this novel screening method for early detection of RA-ILD. In the future, a multicenter study with a larger cohort and a seronegative comparison group would be useful to further substantiate the clinical relevance.

The selected combined threshold value based on pathological findings in lung ultrasound (LUS) and impaired lung function (FVC or DLCO ≤ 80%) is based on a pragmatic, clinically oriented approach. This was defined in the study protocol to enable the most valid screening possible for early-stage RA-ILD, also taking into account the interdisciplinary perspectives in ILD diagnostics.

While rheumatology guidelines increasingly include DLCO as a potential parameter for early lung involvement [8], pulmonology guidelines focus more on FVC as an objective measure. Our results from the ROC analyses support the approach that FVC has lower diagnostic specificity compared to DLCO. Nevertheless, DLCO measurement is known to be susceptible to interindividual variations and dependent on patient cooperation, which limits its informative value, especially in the screening of asymptomatic patients [22, 23].

We therefore opted for a combined examination method in which, as our results show, LUS provides the majority of the diagnostic information, while the additional consideration of DLCO only enables additional discrimination.

This would argue in favor of a possible future simplification of the examination protocol, for example in favor of LUS-based primary screening with targeted functional diagnostics in a second step. However, this hypothesis should be further investigated in larger, multicenter cohorts with multivariate adjustment. Our results clearly show the advantage of the combined approach (see test quality criteria Fig. 2 and Appendix C).

As another limitation, we acknowledge that no formal assessment of interobserver reliability was performed for lung ultrasound (LUS) interpretation. Although two of the three investigators conducted the examinations and image evaluations in consensus to enhance consistency, the absence of quantitative reliability testing represents a limitation of our study. Future work should include structured reproducibility analyses to further substantiate the validity of the LUS findings.

This study employed an additional and non-radiation screening approach using PFT and LUS. While HRCT is considered the gold standard for imaging interstitial lung disease (ILD), it was not routinely performed but rather recommended only for patients with abnormal screening results or with high packyears as tumor screening. Consequently, direct comparison with HRCT findings was possible in only a limited but still relevant subset of 21.2% of all patients (*n* = 43).

A potential verification bias must be acknowledged, as HRCT was performed primarily in patients with an indication based on either high cumulative smoking exposure or screening findings suggestive of ILD. This selective verification could theoretically lead to an overestimation of sensitivity, since false-negative screening results among non-verified patients would remain undetected. However, it should also be noted that these non-verified participants generally had no clinical or sonographic signs of ILD and fewer packyears, making the presence of undiagnosed ILD in this subgroup less likely. Within the HRCT subcohort, only a single case of ILD that was later confirmed during follow-up had been missed by the initial screening. Therefore, while some inflation of sensitivity cannot be excluded, the direction and magnitude of this bias are probably limited in our study population.

In our study, HRCT examinations without contrast agents were requested in order to ensure the validity of the pulmonary assessment. Only three patients underwent HRCT with contrast medium; all other scans were performed without contrast medium. This ensures the diagnostic quality of the HRCT data for the majority of the cases examined. Nevertheless, inconsistent imaging represents a limitation that should be standardized in future prospective studies.

As the HRCT subcohort consists not only of patients with suspected ILD but also of those selected based on high cumulative smoking exposure, the results of the multivariable logistic regression analysis—to identify independent predictors of ILD on HRCT and to assess whether LUS remains significant after adjustment—must be interpreted with caution. Furthermore, the apparently strong discriminatory effect of LUS, reflected by quasi-complete separation and non-convergent estimates, could not be reliably quantified using standard logistic regression methods.

Furthermore, the data represent initial findings from a monocentric study, introducing potential selection and detection biases. To mitigate this, the patients meeting the inclusion criteria were consecutively enrolled, and LUS imaging was independently evaluated by two investigators blinded to additional results.

This study was conducted during the COVID pandemic. We did not receive information from each patient about how often and when each individual had had COVID. There are also some cases in which unclear illnesses with COVID-typical symptoms were reported, but no test for COVID was carried out. Therefore, we cannot say whether past COVID infections had an influence on the results of our study, even if we could not detect a direct impact.

Additionally, data on disease activity (primarily scores and laboratory parameters) were collected only at the time of inclusion. Therefore, no information is available regarding the peak RA activity in each patient. As a result, it is not possible to determine whether a patient experienced a prolonged period of moderate inflammation or an early phase of high disease activity. The same limitation applies to medical history, as no data were collected on how many different treatments were attempted before achieving remission.

As there are no long-term data regarding the consequences of these findings and the development of RA-ILD, future investigation is required. Supporting this, as a multinational cross-sectional study, the ANCHOR-RA study protocol on screening for RA-associated ILD is very promising, using PFT and HRCT as well as LUS and identifying risk factors [31].

To decide which patients are most likely to be affected by RA-ILD, in this context, another part of our project group is working on creating a questionnaire for clinical practice to routinely identify patients with pulmonary pathology. The questionnaire is completed independently by the patients and is based on questions regarding their daily functioning. The patients detected by this questionnaire are then screened for lung diseases [32].

Our study contributes to the growing body of evidence supporting the use of LUS for screening RA-associated ILD. We propose that LUS, in conjunction with PFT, serves as a suitable imaging tool for ILD screening in RA. Our findings are consistent with the ACR/CHEST SARD-ILD guidelines published in 2023–2024, which do not yet include lung ultrasound (LUS) as a recommended method. Our data show that LUS, especially in combination with pulmonary function testing, can be a practical and sensitive approach to early diagnostic screening.

Furthermore, our findings are consistent with the concept of the abovementioned ANCHOR-RA study, which pursues a risk factor-based indication for HRCT. In this context, LUS could serve as an upstream, low-threshold screening method to make the selection of suitable patients for further imaging more targeted.

## Conclusion

**Fig. 4:**
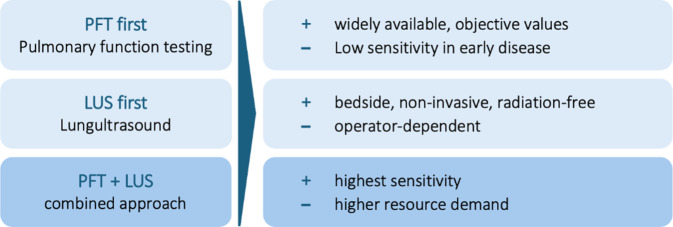
Comparison of the advantages and disadvantages of individual screening methods when used alone or in combination

We recommend a combined screening approach involving pulmonary function testing (PFT) and lung ultrasound (LUS). The combination of both methods in particular highlights the diagnostic strength of our screening protocol and contributes to better identification of patients with subclinical ILD (see test quality criteria; PFT alone: sensitivity 0.86, specificity 0.1; LUS alone: sensitivity 1.0, specificity 0.59; PFT + LUS: sensitivity 0.93, specificity 0.72). For an overview, refer to Figs. 2 and 4 as well as Appendix C for more details.

Our results show that parallel use of PFT and LUS largely compensates for the respective limitations of the individual methods and significantly improves the diagnostic test quality criteria. A comparative schematic representation of different screening pathways (e.g., PFT-first, LUS-first, combined) would be a useful addition to future work in order to further clarify the clinical applicability and relevance for everyday clinical practice.

## Conclusion for practice

- Screening asymptomatic rheumatoid factor (RF)- and anti-citrullinated protein antibody (ACPA)-positive patients for interstitial lung disease (ILD) using lung ultrasound (LUS) and pulmonary function testing (PFT) identifies changes in 16% (suspected ILD).
- An ILD suspicion could be confirmed in 13 cases using high-resolution computed tomography (HRCT; 62%).
- The screening procedure with PFT and LUS has a sensitivity of 93% and a specificity of 72%.
- Screening is easy to perform and without radiation exposure; it is less expensive and more readily available than HRCT.
- The screening is promising, especially for recognizing patients without symptoms but with a risk profile at an early stage in the clinical routine and thus for promoting a better outcome

## Supplementary Information

**A) Exclusion criteria**: The exclusion criteria used in the study are listed below. **B.1) Descriptive representation of the cohort**: The parameters that describe the cohort in more detail are listed below as a mean value with standard deviation or as a number (*n*) and the confidence interval. **B.2) Descriptive representation of the examination results**: The complete examination results are listed below as a mean value with standard deviation or as a number (*n*) and the confidence interval. **C) 2x2 Tables of each screening method inkl. CI95%**: Attached you will find the 2x2 tables for the diagnostic performande of respective screening methods (PFT alone, LUS alone, and the combination of PFT and LUS) as well as the sensitivity, specificity, positive predictive value, and negative predictive value, including the 95% confidence intervals. **D) 2x2 Tables of each screening method inkl. CI95%**: Attached you will find the 2x2 tables showing the diagnostic performance of the respective cut-off values for FVC and DLCOc (initially defined and optimized cut-off value) as well as their sensitivity, specificity, positive predictive value, and negative predictive value, including the 95% confidence intervals.The results are presented for the total cohort and for the CT sub-cohort. **E) Flow diagram: Process of the study**: This flow diagram shows the study process schematically and in the order in which it was carried out.

## Data Availability

Data are included as electronic supplementary material.

## Abbreviations

ACPA: Anti-citrullinated protein antibody
AUC: Area under the curve
95%CI: 95% Confidence interval
DLCOc: Diffusing capacity of the lung for carbon monoxide
EULAR/ACR: European Alliance of associations for Rheumatology/American College of Rheumatology
FVC: Forced vital capacity
HRCT: High-resolution computed tomography
ILD: Interstitial lung disease
Ind.: Indication
LUS: Lung ultrasound
nonILD: No suspicion of ILD
PFT: Pulmonary function testing
PNV: Negative predictive value
PPV: Positive predictive value
RA: Rheumatoid arthritis
RA-ILD: Rheumatoid arthritis-associated interstitial lung disease
RF: Rheumatoid factor
ROC: Receiver operating characteristic
susILD: Suspected ILD

## Declarations

All described studies on patients were carried out with the approval of the responsible ethics committee, in accordance with national law and in accordance with the Declaration of Helsinki of 1975 (in the current, revised version). A declaration of consent has been obtained from all patients involved. Trial registration: German Register of Clinical Studies (DRKS00028871).
