# Supplementary material for: Early detection of RA‑ILD—A novel screening protocol with pulmonary function testing and lung ultrasound: A monocentric cohort study
Source: Z Rheumatol. 2026 Jan 23;85(3):200–13. doi: 10.1007/s00393-025-01775-0 (PMC13021688; doi:10.1007/s00393-025-01775-0)

Early detection of RA-ILD - a novel screening protocol with PFT and LUS:
A monocentric cohort study

Appendix

A) Exclusion criteria

Exclusion criteria will be added as listed below (according to the exclusion criteria of the INBUILD® trial):
Excluding patients with underlying other comorbidities (e.g. restrictive versus obstructive lung function impairment (Tiffeneau Index), pulmonary hypertension / vascular diseases) are considered as given below.

- diagnosis of idiopathic pulmonary fibrosis (IPF)
- creatinine clearance <30 ml/min calculated by Cockcroft-Gault formula at entry into the study
- patients with underlying chronic liver disease (Child Pugh A, B or C hepatic impairment)
- previous treatment with nintedanib or pirfenidone
- other investigational therapy received within 1 month or 6 half-lives (whichever was greater) prior to entry into the study
- significant pulmonary arterial hypertension defined by any of the following:
  - previous clinical or echocardiographic evidence of significant right heart failure
  - history of right heart catheterization showing a cardiac index <= 2 $l/min/m^2$
  - pulmonary arterial hypertension requiring parenteral therapy with epoprostenol / treprostinil
  - primary obstructive airway physiology (pre-bronchodilator FEV1/FVC < 0.7 at entry into the study; Tiffeneau Index)
- in the opinion of the Investigator, other clinically significant pulmonary abnormalities
- major extrapulmonary physiological restriction (e.g. chest wall abnormality, large pleural effusion)
- significant cardiovascular diseases, any of the following:
  - severe hypertension, uncontrolled under treatment (≥160/100 mmHg), within 6 month of entry into the study
  - myocardial infarction within 6 months of entry into the study
  - unstable cardiac angina within 6 months of entry into the study
- other disease that may interfere with testing procedures or in the judgment of the investigator may interfere with trial participation or may put the patient at risk when participating in this trial
- life expectancy for disease other than ILD < 2.5 years (investigator assessment)
- planned major surgical procedures
- women who are pregnant
- patients not able to understand or follow trial procedures including completion of self-administered questionnaires without help
- in the opinion of the investigator, active alcohol or drug abuse

B.1) Descriptive representation of the cohort

as a mean value with standard deviation or as a number (n) and the confidence interval

		total cohort			susILD-group			non-LD-group			CT - subcohort		
		n=203			n=32			n=171			n=43		
Anamnestic data													
**Age**	*years*	59.2	± 12.2	[57.5; 60.9]	64.3	± 12.9	[59.7; 69.0]	58.3	± 11.9	[56.5; 60]	62.7	± 10.9	[59.3; 66.1]
**Sex, female**	*n (%)*	154	75.9%		23	71.9%		131	76.6%		32	74.4%	
**Sex, male**	*n (%)*	49	24.1%		9	28.1%		40	23.4%		11	25.6%	
**BMI**	*kg/m2*	25.7	± 4.6	[25.0; 26.3]	26.1	± 4.5	[24.4; 27.7]	25.6	± 4.7	[24.9; 26.3]	25.4	± 4.8	[23.9; 26.9]
**Smoking, never**	*n (%)*	115	56.7%		12	37.5%		103	60.2%		15	34.9%	
**Smoking, previous**	*n (%)*	54	26.6%		10	31.3%		44	25.7%		15	34.9%	
**Smoking, current**	*n (%)*	34	16.7%		10	31.3%		24	14.0%		13	30.2%	
**Packyears**	*years*	10.7	± 16.3	[8.4; 12.9]	20.2	± 20.6	[12.7; 27.6]	8.9	± 14.8	[6.7; 11.1]	19.9	± 20.4	[13.6; 26.1]
**Disease duration (RA)**	*years*	8.3	± 7.2	[7.3; 9.3]	8.8	± 6.7	[6.4; 11.2]	8.2	± 7.3	[7.1; 9.3]	8.5	± 7.1	[6.3; 10.7]
**Erosive**	*n (%)*	64	31.5%		18	56.3%		46	26.9%		23	53.5%	
**csDMARDs**	*n (%)*	122	60.1%		18	56.3%		104	60.8%		29	67.4%	
**tsDMARDs**	*n (%)*	36	17.7%		3	9.4%		33	19.3%		6	14.0%	
**bDMARDs**	*n (%)*	101	49.8%		18	56.3%		83	48.5%		23	53.5%	
**Steroids**		21	10.3%		3	9.4%		18	10.5%		6	14.0%	
Scores testing the activity of the RA													
**DAS28 CRP**		2.3	± 0.9	[2.1; 2.4]	2.4	± 0.9	[2.1; 2.8]	2.2	± 0.9	[2.1; 2.3]	2.5	± 0.9	[2.2; 2.7]
**DAS28 Score**		2.4	± 1.1	[2.2; 2.5]	2.6	± 1.1	[2.1; 3.0]	2.3	± 1.1	[2.2; 2.5]	2.6	± 1.1	[2.3; 3.0]
**RADAI**		1.9	± 1.5	[1.7; 2.1]	2	± 1.3	[1.6; 2.5]	1.9	± 1.5	[1.7; 2.2]	2.4	± 1.6	[1.9; 2.9]
**RAID**		2.4	± 2.0	[2.1; 2.6]	2.5	± 1.7	[1.9; 3.1]	2.3	± 2	[2.1; 2.6]	2.8	± 1.8	[2.2; 3.3]
**CDAI**		5.7	± 6.2	[4.8; 6.5]	6.8	± 6.1	[4.6; 9.1]	5.5	± 6.2	[4.5; 6.4]	7.7	± 7.4	[5.4; 10.0]
**SDAI**		6.2	± 6.3	[5.3; 7.1]	7.3	± 6.2	[5.1; 9.6]	6	± 6.3	[5.0; 6.9]	8.2	± 7.5	[5.9; 10.5]
Laboratory parameter													
**ERS**	*mm/h*	16.4	± 13.6	[14.5; 18.3]	16.7	± 12	[12.4; 21.1]	16.3	± 13.9	[14.2; 18.4]	16.8	± 14.3	[12.4; 21.2]
**CRP**	*mg/l*	3.9	± 4.4	[3.9; 5.1]	4.8	± 3.5	[3.5; 6.1]	4.4	± 4.6	[3.7; 5.1]	4.4	± 3.2	[3.4; 5.3]
**Rheumafactor**		128.9	± 190.9	[102.5; 155.3]	108.3	± 101.6	[71.7; 144.9]	132.8	± 203.3	[102.1; 163.5]	141.8	± 213.1	[76.2; 207.4]
**CCP-antibodies**		208.0	± 108.3	[193.0; 223.0]	211.5	± 113.2	[170.7; 252.4]	207.3	± 107.7	[191.0; 223.5]	215.8	± 113.0	[181.0; 250.6]
**Creatinine**		0.8	± 0.2	[0.79; 0.84]	0.8	± 0.2	[0.78; 0.88]	0.8	± 0.1	[0.79; 0.85]	0.8	± 0.2	[0.8; 0.9]
**Haemoglobine**		13.9	± 1.1	[13.8; 14.1]	13.9	± 1.2	[13.5; 14.4]	13.9	± 1.1	[13.8; 14.1]	13.8	± 1.0	[13.5; 14.2]
**BNP**		129.0	± 142.4	[109.3; 148.7]	193.0	± 171.2	[131.2; 254.7]	117.0	± 133.6	[96.9; 137.2]	142.1	± 140.3	[98.9; 185.2]

B.2) Descriptive representation of the examination results

as a mean value with standard deviation or as a number (n) and the confidence interval

		total cohort			susILD-group			non-LD-group			CT - subcohort		
		n=203			n=32			n=171			n=43		
Lufu / Lung function testing													
**FVC %**	%	97.3	± 16.9	[95.0; 99.7]	94.6	± 16.2	[88.8; 100.5]	97.9	± 17.0	[95.3; 100.4]	94.7	± 19.1	[88.9; 100.6]
**FEV1 %**	%	95.3	± 18.1	[92.8; 97.8]	90.8	± 19	[83.9; 97.6]	96.2	± 17.9	[93.5; 98.9]	87.4	± 20.9	[81.0; 93.9]
**DLCOc %**	%	80.2	± 15.6	[78.0; 82.4]	68.2	± 13.5	[63.3; 73.1]	82.4	± 14.9	[80.2; 84.7]	70.5	± 15.8	[65.7; 75.4]
**Tiffenau**		0.8	± 0.1	[0.77; 0.79]	0.8	± 0.1	[0.7; 0.8]	0.8	± 0.1	[0.8; 0.8]	0.7	± 0.1	[0.7; 0.8]
**TLC**	%	105.7	± 14.8	[103.7; 107.7]	105.1	± 13.8	[100.1; 110.1]	105.8	± 15.0	[103.6; 108.1]	104.2	± 15.1	[99.6; 108.9]
**RV**	%	117.5	± 32.4	[113.0; 122.0]	121.6	± 27.8	[111.6; 131.7]	116.7	± 33.2	[111.7; 121.7]	126.1	± 37.5	[114.4; 137.8]
CPET / Cardiopulmonary exercise testing													
**Peak work rate**		127.8	± 44.5	[121.5; 134.0]	109.6	± 36.4	[96.5; 122.7]	131.3	± 45.2	[124.4; 138.3]	110.8	± 37.9	[99.2; 122.5]
**Predicted work rate**		120.3	± 37.1	[115.1; 125.5]	105.1	± 41.1	[90.3; 119.9]	123.2	± 35.6	[117.8; 128.7]	109.3	± 38.0	[97.6; 121.0]
**CPET VO2peak**	%	97.3	± 23.7	[93.9; 100.7]	86.9	± 23.0	[78.5; 95.4]	99.3	± 23.4	[95.7; 102.9]	91.6	± 23.0	[84.3; 98.8]
**CPET VO2 AT**	%	79.5	± 23.0	[76.1; 82.8]	71.8	± 22.5	[63.4; 80.2]	81.0	± 22.9	[77.3; 84.7]	76.9	± 22.7	[69.3; 84.5]
**EQCO2 Slope**		29.6	± 6.1	[28.8; 30.5]	32.3	± 5.5	[30.3; 34.3]	29.1	± 6.1	[28.2; 30.1]	31.6	± 5.9	[29.7; 33.5]
**pO2 peak**		84.7	± 11.8	[83.0; 86.4]	82.7	± 13.1	[77.8; 87.6]	85.1	± 11.6	[83.3; 86.9]	83.1	± 13.5	[78.8; 87.5]
**pCO2 peak**		35.5	± 4.6	[34.9; 36.2]	34.1	± 5.6	[32.0; 36.2]	35.8	± 4.4	[35.1; 36.5]	35.1	± 5.3	[33.3; 36.8]
LUS / Lung ultrasound													
**LUS sPAP**		24.1	± 6.9	[23.1; 25.1]	26.5	± 7.5	[23.5; 29.5]	23.8	± 6.7	[22.7; 24.8]	25.3	± 7.4	[22.9; 27.7]
**LUS Tapse**		24.0	± 3.8	[23.5; 24.6]	24.4	± 3.9	[22.8; 26.0]	24.0	± 3.8	[23.4; 24.6]	24.2	± 3.6	[22.9; 25.4]
**LUS LV_EF**		65.8	± 6.1	[64.9; 66.7]	67.6	± 3.9	[66.2; 69.0]	65.5	± 6.4	[64.4; 66.5]	65.7	± 5.5	[64.0; 67.5]
Evaluation Lunfunctionntests													
**Abnormalities in total**	*n (%)*	82	40.4%		30	93.8%		52	31.1%		33	78.6%	
**Diffusion disorder**	*n (%)*	58	28.6%		27	84.4%		31	18.6%		27	64.3%	
**Restriction**	*n (%)*	13	6.4%		2	6.3%		11	6.6%		4	9.5%	
**Hyperinflation**	*n (%)*	8	3.9%		2	6.3%		6	3.6%		4	9.5%	
**Obstruction**	*n (%)*	26	12.8%		9	28.1%		17	10.2%		14	33.3%	
**limited FVC**	*n (%)*	2	1.0%		2	6.3%		0	0.0%		1	2.4%	
**postOP gentle respiration**	*n (%)*	3	1.5%		1	3.1%		2	1.2%		1	2.4%	
Evaluation Lung ultrasound													
**Abnormalities in total**	*n (%)*	107	52.7%		32	100.0%		75	44.1%		36	83.7%	
**Pleural effusion**	*n (%)*	14	6.9%		4	12.5%		10	5.9%		5	11.6%	
**ILD**	*n (%)*	58	28.6%		31	96.9%		27	15.9%		26	60.5%	
**focal changes/tumor**	*n (%)*	16	7.9%		3	9.4%		13	7.6%		7	16.3%	
**congestions**	*n (%)*	2	1.0%		0	0.0%		2	1.2%		0	0.0%	
**pleural irregularities**	*n (%)*	26	12.8%		0	0.0%		26	15.3%		4	9.3%	
**others**	*n (%)*	5	2.5%		1	3.1%		4	2.4%		1	2.3%	
**comet tail artefacts**	*n (%)*	58	28.6%		22	68.8%		36	21.2%		19	44.2%	
**B-lines**	*n (%)*	5	2.5%		3	9.4%		2	1.2%		2	4.7%	
Evaluation ILD suspicion													
**Based on LUS**	*n (%)*	65	32.0%		31	96.9%		34	20.0%		26	60.5%	
**Based on Lufu and LUS**	*n (%)*	32	15.8%		32	100.0%		171	100.0%		21	48.8%	

C) 2x2 Tables of each screening method incl. CI95%

PFT & LUS	**ILD** (diagnosed by HRCT)	**non-ILD** (diagnosed by HRCT)	
**ILD suspicion** (by screening)	13<br>30.23%	8<br>18.60%	21<br>48.84%
**non-ILD suspicion** (by screening)	1<br>2.33%	21<br>48.84%	22<br>51.16%
	14<br>32.56%	29<br>67.44%	43<br>100%

**Diagnostic performance**		95%-CI
Sensitivity	0.93	0.69 ; 0.99
Specificity	0.72	0.54 ; 0.85
PPV	0.62	0.41 ; 0.79
NPV	0.95	0.78 ; 0.99

LUS alone	**ILD** (diagnosed by HRCT)	**non-ILD** (diagnosed by HRCT)	
**ILD suspicion** (by screening)	14<br>32.56%	12<br>27.91%	26<br>60.47%
**non-ILD suspicion** (by screening)	0<br>0.00%	17<br>39.53%	17<br>39.53%
	14<br>32.56%	29<br>67.44%	43<br>100%

**Diagnostic performance**		95%-CI
Sensitivity	1.00	0.78 ; 1.00
Specificity	0.59	0.41 ; 0.74
PPV	0.54	0.35 ; 0.71
NPV	1.00	0.82 ; 1.00

PFT alone	**ILD** (diagnosed by HRCT)	**non-ILD** (diagnosed by HRCT)	
**ILD suspicion** (by screening)	12<br>27.91%	26<br>60.46%	38<br>88.37%
**non-ILD suspicion** (by screening)	2<br>4.65%	3<br>6.98%	5<br>11.63%
	14<br>32.56%	29<br>67.44%	43<br>100%

**Diagnostic performance**		95%-CI
Sensitivity	0.86	0.60 ; 0.96
Specificity	0.10	0.04 ; 0.26
PPV	0.32	0.19 ; 0.47
NPV	0.60	0.23 ; 0.88

D) 2x2 Tables of each screening method incl. CI95%

Total cohort (n=203) / initially defined Cut-Off of ≦ 80%

FVC % ≦ 80%	sus-ILD	non-ILD	
Test positive	7<br>3.45%	23<br>11.33%	30<br>14.78%
Test negative	25<br>12.32%	148<br>72.91%	173<br>85.22%
	32<br>15.76%	171<br>84.24%	203<br>100%

Diagnostic performance		95%-CI
Sensitivity	0.22	0.11 ; 0.39
Specificity	0.87	0.81 ; 0.91
PPV	0.23	0.12 ; 0.41
NPV	0.86	0.80 ; 0.90

DLCOc % ≦ 80%	sus-ILD	non-ILD	
Test positive	26<br>12.81%	69<br>33.99%	95<br>46.80%
Test negative	6<br>2.96%	102<br>50.25%	108<br>53.20%
	32<br>15.76%	171<br>84.24%	203<br>100%

Diagnostic performance		95%-CI
Sensitivity	0.81	0.65 ; 0.91
Specificity	0.60	0.52 ; 0.67
PPV	0.27	0.19 ; 0.37
NPV	0.94	0.88 ; 0.97

Total cohort (n=203) / optimized Cut-Off with Youden-Index

FVC % ≦ 85.5%	sus-ILD	non-ILD	
Test positive	11<br>5.42%	29<br>14.29%	40<br>19.70%
Test negative	21<br>10.34%	142<br>69.95%	163<br>80.30%
	32<br>15.76%	171<br>84.24%	203<br>100%

Diagnostic performance		95%-CI
Sensitivity	0.34	0.20 ; 0.52
Specificity	0.83	0.77 ; 0.88
PPV	0.28	0.16 ; 0.43
NPV	0.87	0.81 ; 0.91

DLCOc % ≦ 79.5%	sus-ILD	non-ILD	
Test positive	25<br>12.32%	62<br>30.54%	87<br>42.86%
Test negative	7<br>3.45%	109<br>53.69%	116<br>57.14%
	32<br>15.76%	171<br>84.24%	203<br>100%

Diagnostic performance		95%-CI
Sensitivity	0.78	0.61 ; 0.89
Specificity	0.64	0.56 ; 0.71
PPV	0.29	0.20 ; 0.39
NPV	0.94	0.88 ; 0.97

CT-subcohort (n=43) / initially defined Cut-Off of ≦ 80%

FVC % ≦ 80%	CT-ILD	CT-nonILD	
Test positive	3<br>6.98%	8<br>18.60%	11<br>25.58%
Test negative	11<br>25.58%	21<br>48.84%	32<br>74.42%
	14<br>32.56%	29<br>67.44%	43<br>100%

Diagnostic performance		95%-CI
Sensitivity	0.21	0.08 ; 0.48
Specificity	0.72	0.54 ; 0.85
PPV	0.27	0.10 ; 0.57
NPV	0.66	0.48 ; 0.86

DLCOc % ≦ 80%	CT-ILD	CT-nonILD	
Test positive	11<br>25.58%	24<br>55.81%	35<br>81.40%
Test negative	3<br>6.98%	5<br>11.63%	8<br>18.60%
	14<br>32.56%	29<br>67.44%	43<br>100%

Diagnostic performance		95%-CI
Sensitivity	0.79	0.52 ; 0.92
Specificity	0.17	0.08 ; 0.35
PPV	0.31	0.19 ; 0.48
NPV	0.63	0.31 ; 0.86

CT- subcohort (n=43) / optimized Cut-Off with Youden-Index

FVC % ≦ 103.5%	CT-ILD	CT-nonILD	
Test positive	2<br>4.65%	27<br>62.79%	29<br>67.44%
Test negative	12<br>27.91%	2<br>4.65%	14<br>32.56%
	14<br>32.56%	29<br>67.44%	43<br>100%

Diagnostic performance		95%-CI
Sensitivity	0.14	0.04 ; 0.40
Specificity	0.07	0.02 ; 0.22
PPV	0.07	0.02 ; 0.22
NPV	0.14	0.04 ; 0.40

DLCOc % ≦ 58%	CT-ILD	CT-nonILD	
Test positive	4<br>9.30%	4<br>9.30%	8<br>18.60%
Test negative	10<br>23.26%	25<br>58.14%	35<br>81.40%
	14<br>32.56%	29<br>67.44%	43<br>100%

Diagnostic performance		95%-CI
Sensitivity	0.29	0.12 ; 0.55
Specificity	0.87	0.69 ; 0.95
PPV	0.50	0.22 ; 0.79
NPV	0.71	0.55 ; 0.84

E) Flow diagram: Process of the study

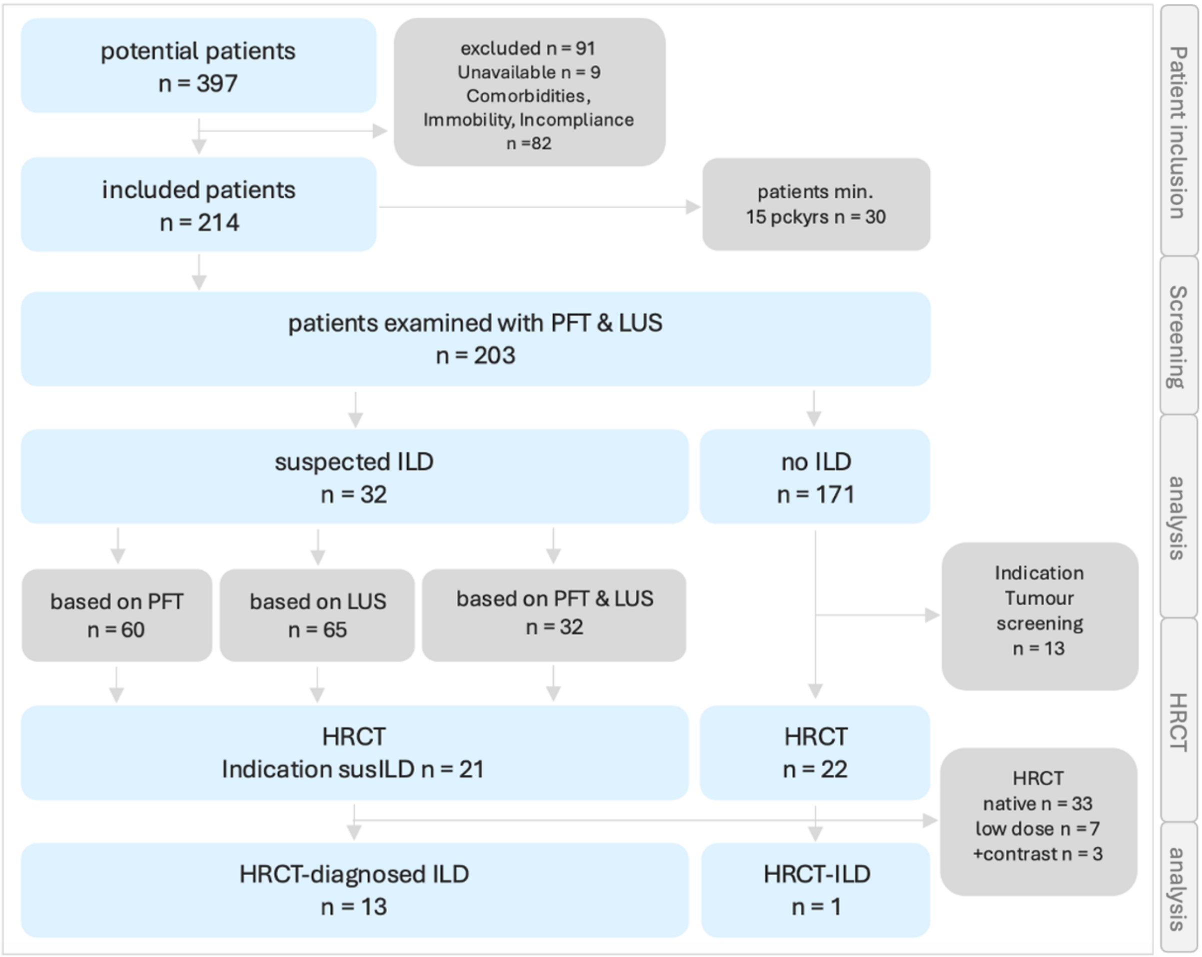

Supplement: Supplementary file 1 — A) Exclusion criteria: The exclusion criteria used in the study are listed below. B.1) Descriptive representation of the cohort: The parameters that describe the cohort in more detail are listed below as a mean value with standard deviation or as a number (n) and the confidence interval. B.2) Descriptive representation of the examination results: The complete examination results are listed below as a mean value with standard deviation or as a number (n) and the confidence interval. C) 2x2 Tables of each screening method inkl. CI95%: Attached you will find the 2x2 tables for the diagnostic performande of respective screening methods (PFT alone, LUS alone, and the combination of PFT and LUS) as well as the sensitivity, specificity, positive predictive value, and negative predictive value, including the 95% confidence intervals. D) 2x2 Tables of each screening method inkl. CI95%: Attached you will find the 2x2 tables showing the diagnostic performance of the respective cut-off values for FVC and DLCOc (initially defined and optimized cut-off value) as well as their sensitivity, specificity, positive predictive value, and negative predictive value, including the 95% confidence intervals.The results are presented for the total cohort and for the CT sub-cohort. E) Flow diagram: Process of the study: This flow diagram shows the study process schematically and in the order in which it was carried out. [file 393_2025_1775_MOESM1_ESM.pdf]
